# Polydopamine-Coated Poly(l‑lactide) Nanofibers with Controlled Release of IL-10 for Effective Management of Peripheral and Central Neuropathic Pain in Rats

**DOI:** 10.1021/acsbiomaterials.5c01638

**Published:** 2025-11-13

**Authors:** Jie Zhao, Tao Xue, Zhiyuan Liu

**Affiliations:** † Department of Orthopedics, Wujin Hospital Affiliated with Jiangsu University, Changzhou 213003, China; ‡ The Wujin Clinical College of Xuzhou Medical University, Changzhou 213003, China; § Changzhou Key Laboratory of Molecular Diagnostics and Precision Cancer Medicine, Changzhou 213003, China

**Keywords:** PDA, PLLA, nanofibers, IL-10, neuropathic pain, neuroinflammation

## Abstract

Managing neuropathic pain is challenging due to the complex pathogenetic mechanisms and limited available drugs and therapeutic options. Innovative nanofiber systems offer a novel method to effectively administer therapeutic cytokines, drugs, or small molecule compounds to achieve better relief of neuropathic pain. Here, we report a novel nanofiber with controlled release of IL-10 for implantation into ligated nerve roots and hemisection-injured spinal cords to exert anti-inflammatory effects and attenuate neuropathic pain. An electrospun poly­(l-lactide) (PLLA) nanofiber was modified by a polydopamine (PDA) coating, and this nanofiber demonstrated sustained release of IL-10 in vitro. We showed that L5 spinal nerve ligation and T13 spinal cord hemisection induced peripheral and below-level central neuropathic pain in rats, along with the development of neuroinflammation. Spinal IL-10 was also markedly changed after spinal nerve ligation (SNL) or spinal cord injury (SCI), especially in the spinal dorsal horn. Rats were treated postsurgically with IL-10 that was loaded on PDA@PLLA nanofibers as an implantable cytokine delivery system. IL-10 levels were significantly upregulated after the implantation of PDA@PLLA nanofibers in SNL and SCI rats. We further demonstrated that the implantation could inhibit glial activation and neuronal excitability in vivo, along with suppressing the expression levels of pro-inflammatory cytokines. Most importantly, IL-10-loaded PDA@PLLA nanofiber treatment attenuated the development and maintenance of peripheral and central neuropathic pain. Together, the combination of IL-10 and PDA@PLLA nanofibers provides a novel implantable delivery system for treating neuroinflammatory conditions and may emerge as a promising agent to prevent chronic neuropathic pain.

## Graphical abstract

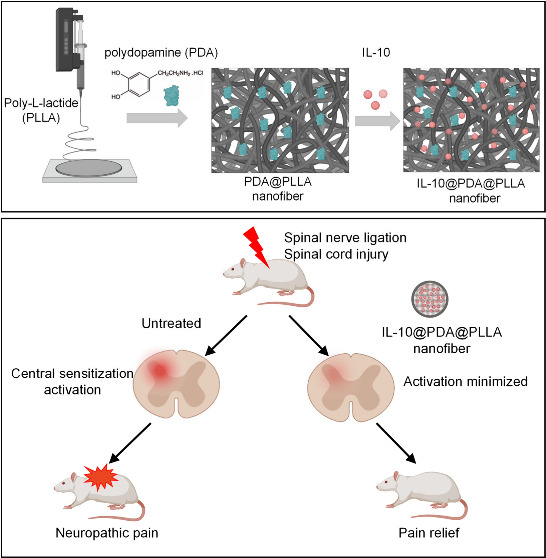

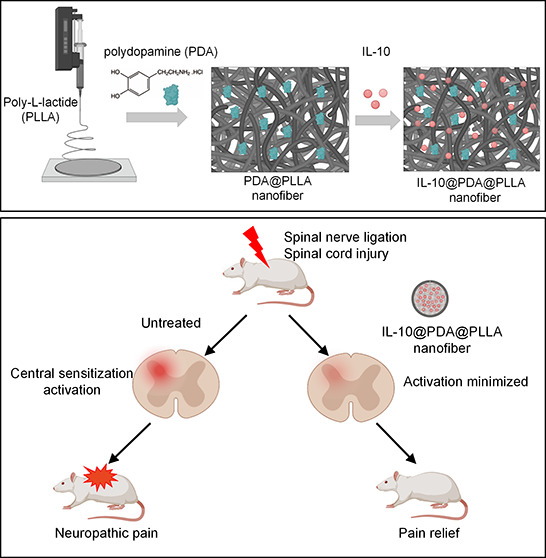

## Introduction

Neuropathic pain is usually directly caused by lesions or diseases of the somatosensory nervous system, including central and peripheral nervous system injuries, surgery, various inflammation and infections, or degenerative diseases of the central nervous system.^1^ Current evidence suggests that almost all patients with spinal cord injury will feel various forms of pain after injury, of which more than half of patients will develop chronic pain that will further hinder the recovery of the disease, bringing serious physical and psychological burdens to patients.^2^ However, available treatment options have a limited effect on neuropathic pain.^3^ Tricyclic antidepressants, 5-hydroxytryptamine, pregabalin, and gabapentin are usually commended as first-line drugs.^4^ Drug therapy works in a minority of patients and has dose-limiting side effects, according to a meta-analysis.^5^ On the other hand, it is hard to prove that physical therapies, such as repetitive transcranial magnetic stimulation^6^ and transcranial direct current stimulation,^7^ have repeatable positive effects on reliving neuropathic pain.

Neuropathic pain is associated with chronic neuroinflammation. Accumulating evidence suggests that certain kinds of cells such as immune cells, glial cells, and stem cells play active roles in the pathogenesis and resolution of pain.^8^ In depth, various pro-inflammatory factors and chemokines work.^9,10^ Cao et al. demonstrated that CXCL1 exerts an antinociceptive effect in neuropathic pain following peripheral nerve injury, which might be mediated by the infiltrating neutrophils.^11^ A study showed that IL-1β contributes to the upregulation of nerve growth factor during inflammation, which plays a major role in the production of inflammatory pain.^12^ Furthermore, TNF-α has effects on hyperalgesic response to inflammation.^13^ Our previous experiments showed similar results that mRNA levels of TNF-α, IL-1β, and IL-6 were significantly upregulated in the lumbar 4/5 segment in a spinal nerve ligation rat model.^14^ IL-4, IL-10, TNF-β, etc., have the opposite effect on inflammatory pain and may be beneficial in the treatment of neuropathic pain.^15–18^

IL-10, as one of the most important anti-inflammatory factors, has the ability to inhibit the secretion of pro-inflammatory factors including TNF-α, IL-6, and IL-1β. The regulatory role of IL-10 and its related mechanisms in chronic pain, covering neuropathic pain, have also been widely studied and discovered.^19–22^ A single intrathecal injection of IL-10 alleviated the hyperalgesia caused by chronic constriction injury (CCI) lesion by blocking the recruitment of macrophages and preventing the production of TNF-α at the injured site.^16^ HSV-mediated IL-10 not only inhibited TNF-α production of microglia but also reduced the phosphorylation of p38 MAPK and ultimately alleviated pain sensitivity.^23^ Wu et al. further found that IL-10 was involved in the regulation of β-endorphin expression in microglia of the spinal cord in addition to its previous inhibitory effect on pro-inflammatory factors and further affected neuropathic pain caused by selective spinal nerve ligation.^22^ CD8^+^ T-cell infiltration upregulated IL-10 receptor expression in dorsal root ganglia and in turn suppressed aberrant spontaneous firing of DRG neurons, facilitating recovery from chemotherapy-induced peripheral neuropathy.^24,25^ In conclusion, IL-10 can regulate neuropathic pain through various mechanisms. However, how to effectively use it to relieve neuropathic pain is still a challenge researchers face.

As an important part of tissue engineering (TE), scaffolds can serve as carriers for the release of various drugs and bioactive factors. Poly­(l-lactide) (PLLA) is a kind of synthetic material with good biodegradability and biocompatibility.^26^ Furthermore, electrospun nanofibrous membranes, as one of the most promising scaffolds, have been proved well-suited for TE.^27^ Considering the affinity between scaffolds and bioactive factors and the rate of factor release, polydopamine (PDA) coatings have been inserted in electrospun nanofibrous membranes, which is a highly advantageous modification to achieve the controlled release of growth factors effectively.^28–31^ In the field of bone regeneration, various kinds of artificial materials loaded with bone morphogenetic protein-2 (BMP-2) proved that PDA coatings promote the slow release of BMP-2.^29,32^ Besides, our past studies have shown that an electrospun PLLA film modified with a PDA coating is a promising scaffold.^33^ Thus, we developed a novel PDA-coated PLLA nanofiber with a controlled release of IL-10 for relieving neuropathic pain.

## Materials and Methods

### Nanofiber Materials

PLLA (33135-50-1) was obtained from Yuanye Biotechnology (Shanghai, China). Dichloromethane (DCM) (D116143) and dimethylformamide (DMF) (D112009) were supplied by Aladdin (Shanghai, China). Dopamine (A11136.06) was purchased from Alfa Aesar (Ward Hill, MA, USA). Tris-HCl (pH 8.5) (ST785) was provided by Beyotime (Shanghai, China).

### Fabrication of Electrospun PLLA Fibers

A total of 500 mg of PLLA was completely dissolved in 3 mL of DCM, followed by the addition of 2.1 mL of DMF with thorough mixing. The resulting solution was loaded into a syringe connected to a syringe pump (Tianjin Dongwen High Voltage Power Supply Corp., Tianjin, China) and subjected to electrospinning. An aluminum-foil-covered rotating drum collector (Suzhou Biaosheng Electromechanical Equipment Co., Ltd., Suzhou, China) served as the anode. Circular glass slides, each with an area of approximately 1.5 cm^2^, were placed on the aluminum foil surface for sample collection. The distance between the syringe tip and collector was maintained at 12 cm, and a voltage of 18 kV was applied between them. The flow rate was set to 1 mL/h, and the collector rotation speed was maintained at 70 rpm.

### PDA Coating

Dopamine deposition onto the PLLA scaffolds was performed by using a direct immersion coating method. The scaffolds were first rinsed with deionized water and then immersed in a dopamine solution (1 mg/mL in 10 mM Tris-HCl buffer, pH 8.5) under gentle shaking at room temperature overnight. Subsequently, the scaffolds were washed three times with deionized water to remove any unbound dopamine residues and for further study.

### Loading of IL-10

The PLLA and PDA@PLLA membranes were sterilized by immersion in 75% ethanol for 3 days and subsequently equilibrated in phosphate-buffered saline (PBS) for 2 days. Before further use, the scaffolds were rinsed three times with PBS. Recombinant rat interleukin-10 (IL-10) protein (Sino Biological, Shanghai, China) was immobilized onto the surfaces of the PLLA and PDA@PLLA membranes via direct infiltration. Specifically, the scaffolds were immersed in an IL-10 solution (800 ng/mL in deionized water) and gently shaken at 4 °C overnight to obtain IL-10@PLLA and IL-10@PDA@PLLA membranes. Finally, all scaffolds were washed three times with deionized water to remove unbound IL-10.

### Morphology Analysis of the Membranes

The surface morphology of the scaffolds was characterized by using a scanning electron microscope (SEM, S-4800; Hitachi, Kyoto, Japan). For Fourier transform infrared spectroscopy (FTIR) analysis, the scaffolds were ground into fine powder and analyzed by using a Nicolet 6700 spectrometer (Thermo Fisher Scientific, Waltham, MA, USA).

### Controlled Release of IL-10

The IL-10@PLLA and IL-10@PDA@PLLA membranes were prepared as described above. Each membrane was immersed in 10 mL of phosphate-buffered saline (PBS) in an Eppendorf (EP) tube and incubated on a thermostatic shaker (MQT-60R; Shanghai Minquan Instrument Co., Ltd., Shanghai, China) at 37 °C and 500 rpm. At predetermined time points (2 h, 6 h, 12 h, 1 d, 2 d, 3 d, 4 d, 5 d, 7 d, 10 d, and 14 d), the PBS was collected and stored at −80 °C, after which 2 mL of fresh PBS was added to maintain the total volume. The IL-10 concentration in each sample was quantified using a commercial ELISA kit according to the manufacturer’s instructions. Absorbance was measured with a spectrophotometer (Synergy MX, BioTek, Winooski, VT, USA). The cumulative release of IL-10 at each time point was then calculated, and the release profile was plotted accordingly.

### Animals

Male Sprague–Dawley rats weighing 200–220 g were used for all experiments. Animals were housed at 24 ± 1 °C under a 12 h light/dark cycle with ad libitum access to standard chow and water. All procedures were approved by the Institutional Animal Care and Use Committee of Jiangsu University.

### Drugs and Antibodies

The recombinant rat protein IL-10 (80082-RNAE) was purchased from Sino Biological (Shanghai, China). Antibodies against IL-10 (82191-3-RR) were purchased from Proteintech (Hubei, Wuhan, China). Antibodies against c-Fos (ab208942) and iba-1 (ab178847) were purchased from Abcam (Cambridge, MA, USA). Antibodies against GFAP (#3670S), β-actin (#4970), mouse IgG (H+L) (#4408), and rabbit IgG (H+L) (#4413) were purchased from CST. Goat anti-rabbit IgG H&L (HRP) (ab6721) and goat anti-mouse IgG H&L (HRP) (ab205719) were purchased from Abcam.

### Spinal Nerve Ligation (SNL)

Lumbar 5 (L5) SNL surgery in rats was performed as previously reported.^34^ Anaesthesia was induced with 10% chloral hydrate (0.8–1.2 mL, i.p.). The left L6 transverse process was resected to visualize the L4 and L5 spinal nerves. The L5 spinal nerve was carefully isolated and tightly ligated with 5-0 silk. After complete hemostasis was confirmed, the wound was closed in layers with 3-0 silk and sterilized with ethanol followed by iodophor. Sham-operated animals underwent identical surgical exposure but without nerve ligation.

### Spinal Cord Injury (SCI)

Spinal cord hemisection surgery was performed according to a method described previously.^35^ Under deep anesthesia, a T10–T11 laminectomy was performed to expose the lumbar enlargement region. A left-sided hemisection of the spinal cord was then made at the T13 segment, preserving the dorsal vasculature. Sham animals received a laminectomy only. Postoperatively, rats were kept normothermic and singly housed with proper monitoring.

### Implantation of PDA@PLLA Nanofibers

For the groups requiring fibrous membrane implantation, the initial procedures were identical to those for the standard SCI and SNL groups. After the L5 spinal nerve was ligated or the T13 spinal cord was subjected to hemisection, the nanofibers (1.5 cm^2^) were implanted before wound closure. The PDA@PLLA nanofibers were wrapped around the ligated spinal nerve in SNL rats, and they were placed on the surface of the hemisectioned spinal cord. Then, the incision was closed with care to minimize fibrous membrane displacement.

### Assessment of Mechanical Pain-Related Behaviors

Nociceptive testing was conducted in compliance with the ethical guidelines set forth by the International Association for the Study of Pain (IASP). The experimenter remained unaware of the specific animal groups and treatments assigned. Consistent experimental conditions were maintained, and the rats were allowed a 30 min acclimation period prior to testing. Baseline measurements were taken 1 day before surgery. Subsequently, the rats were randomly assigned to different treatment groups. Mechanical stimulation was applied three times to each hind paw, with 5 min intervals between stimulations, and each group consisted of six rats.

Mechanical nociception was quantified with an electronic Von Frey system (e-VF, 38450, Ugo Basile, Italy). A rigid metallic probe was applied to the plantar hind paw, and the force ramped linearly at a consistent rate of 10 g/s until a brisk withdrawal occurred. The device automatically captured the peak force (paw-withdrawal threshold, PWT) at the moment of reflexive removal.

For the assessment of pain-related behavior after SNL, tests were performed at the following time points after surgery: 3, 5, 7, 10, 14, and 21 days.

Additionally, to evaluate pain-related behavior following SCI, tests were conducted at various time points postsurgery, specifically at 5, 7, 10, 14, 21, 28, and 35 days.

To determine the effect of the IL-10-releasing PDA@PLLA nanofibrous membrane on the SNL-induced peripheral neuropathic pain and SCI-induced central neuropathic pain, pain behavior tests were conducted at the same time points as mentioned above.

### Immunohistochemistry

L4/5 spinal cord segments were prepared at appropriate time points.

To explore the distribution and expression profiles of IL-10 in the superficial dorsal horn (SDH) after SNL and SCI, spinal segments were collected 7 or 10 days after surgery. Spinal segments were harvested 7 days after SNL or 10 days after SCI to assess the role of the implantation of IL-10-releasing PDA@PLLA nanofibrous membranes in neuronal excitability and glial activation.

Following deep anesthesia, animals were transcardially perfused with 0.9% saline followed by 4% paraformaldehyde. The L4–L5 spinal segment was rapidly dissected, postfixed overnight, and cryoprotected in ascending sucrose solutions (20% and 30% in PBS). Serial transverse cryosections (10 μm) were cut for immunofluorescence staining. The sections were incubated with primary antibodies: rabbit anti-IL-10 (1:500, Proteintech), mouse anti-GFAP (astrocytic marker, 1:1000, CST), rabbit anti-iba-1 (microglial marker, 1:500, Abcam), and mouse anti-c-Fos (neuronal activity marker, 1:1500, Abcam). After that, sections were incubated with the appropriate Alexa Fluor 488- or 555-conjugated secondary antibodies (1:1000, CST).

Images were acquired on a Nikon Ni-E fluorescence microscope and analyzed with ImageJ (NIH Image, Bethesda, MD, USA). Semiquantitative analysis was performed using three representative images from three independent experiments, which included the entire superficial dorsal horn (SDH) in each group.

### Real-Time PCR

Spinal cord tissue was harvested at 1, 3, 5, 7, 10, and 14 days postinjury for quantitative mRNA analysis of pro-inflammatory cytokines (TNF-α, IL-1β, and IL-6) and anti-inflammatory cytokines (IL-10 and IL-4).

After rapid decapitation, the L4–L5 spinal segment was removed and subsequently divided into ipsilateral and contralateral halves. Total RNA was isolated with TRIzol reagent (Takara, Shiga, Japan) and reverse-transcribed using a PrimeScript RT kit (Takara, Shiga, Japan). The cDNA was diluted and amplified with gene-specific primers (Table 1) in an iTaqTM Universal SYBR Green Supermix (Bio-Rad) on an ABI 7500 Fast system (Applied Biosystems, Foster City, CA, USA). Cycling parameters were as follows: 95 °C for 15 s, followed by 35 cycles of 95 °C for 10 s and 60 °C for 30 s, ending with 95 °C for 15 s. For each gene, three replicate wells were utilized, and the entire assay was repeated three times. Relative mRNA expression levels were calculated by using the comparative CT method.

### Western Blot Analysis

For temporal profiling of IL-10 protein after SNL or SCI, spinal cords were obtained at 1, 3, 5, 7, 10, 14, and 21 days post-SNL and at 3, 7, 10, 14, 21, 28, and 35 days post-SCI. To explore the effects of the IL-10-releasing PDA@PLLA nanofibrous membrane on IL-10 expression, segments were collected at 7 and 14 days after SNL and SCI. Samples were homogenized in ice-cold RIPA buffer. After the measurement of protein concentration, the tissue or cell protein samples were separated via SDS-PAGE and transferred onto PVDF membranes. Protein samples were then incubated overnight at 4 °C with rabbit anti-IL-10 (1:1000; Proteintech) and mouse antiactin (1:2000; CST) antibodies, followed by HRP-conjugated secondary antibodies. Immunoreactive bands were visualized by using a CLINX imaging system (CLINX Science Instrument, Shanghai, China). The protein intensities were quantified with ImageJ software (NIH Image, Bethesda, MD, USA).

### Enzyme-Linked Immunosorbent Assay (ELISA)

To determine the effect of the IL-10-releasing PDA@PLLA nanofibrous membrane on neuroinflammation following SNL and SCI, the spinal cord was collected 10 days after surgery and implantation. The ipsilateral L4–L5 spinal cord was rapidly removed, minced on ice, and homogenized in ice-cold PBS. The homogenate was cleared by centrifugation (13 000 × g, 10 min, 4 °C) and the supernatant retained. Total protein concentration was quantified with a BCA assay kit (Beyotime, Shanghai, China). TNF-α, IL-1β, and IL-6 concentrations were determined with ELISA kits (Solarbio, Beijing, China; No. SEKR-0009, SEKR-0002, and SEKR-0005, respectively), used strictly according to the manufacturer’s instructions.

### Statistical Analysis

Statistical analyses were performed with GraphPad Prism 9.5 (GraphPad Software, Inc., San Diego, CA, USA). Western blot, IHC, real-time PCR, and ELISA data were evaluated by one-way analysis of variance (ANOVA) followed by Dunnett’s multiple comparison test. Behavioral outcomes were analyzed using two-way ANOVA with repeated measures followed by Bonferroni’s multiple comparisons test. All data are presented as the mean ± SEM. *p* < 0.05 was considered statistically significant.

## Results

### Characteristics of the Nanofibers

As shown in Figure 1A, the electrospun PLLA membrane displayed a highly interconnected, porous nanofibrous network, perfectly matching the morphology described in the literature. This provided the possibility for the subsequent loading of IL-10 on all kinds of PLLA membranes, which are considered as commonly used scaffolds in tissue engineering. After polydopamine (PDA) modification, a conformal PDA layer became readily apparent on the PLLA surface in the SEM images (Figure. [Fig fig1]B1), confirming successful surface functionalization. In addition, IL-10 could not be directly observed in IL-10@PLLA and IL-10@PDA@PLLA samples because a single IL-10 cytokine molecule was below the SEM resolution limit. However, we observed the PDA deposition layer under SEM at a higher magnification (Figure. [Fig fig1]B2). Complementary Fourier transform infrared (FTIR) spectroscopy provided unambiguous comforting proof of IL-10 loading, as shown in Figure. [Fig fig1]C. New amide I (∼1650 cm^–1^) and amide II (∼1540 cm^–1^) absorption bands appear in both IL-10-loaded membranes, while the PDA-coated variant shows markedly stronger band intensities, reflecting its superior affinity and higher loading capacity for IL-10. It can be seen that the PLLA membrane could load IL-10 through simple adsorption, although the ability seems too weak, which we speculated is related to the hydrophilicity of IL-10. The PDA coating was able to not only retain the integrity of the PLLA film skeleton but also enhance its loading capacity by improving the hydrophobicity of the PLLA membrane. Although there was no direct evidence found by SEM, the FTIR results indicated that the PDA coating, as a kind of adhesive layer, had a strong secondary modification ability. The above results indicated that PDA functionalization not only preserves the intrinsic microporous architecture of electrospun PLLA membrane but also significantly enhances its ability to immobilize and stabilize therapeutic cytokines for potential controlled-release applications.

**Figure 1:**
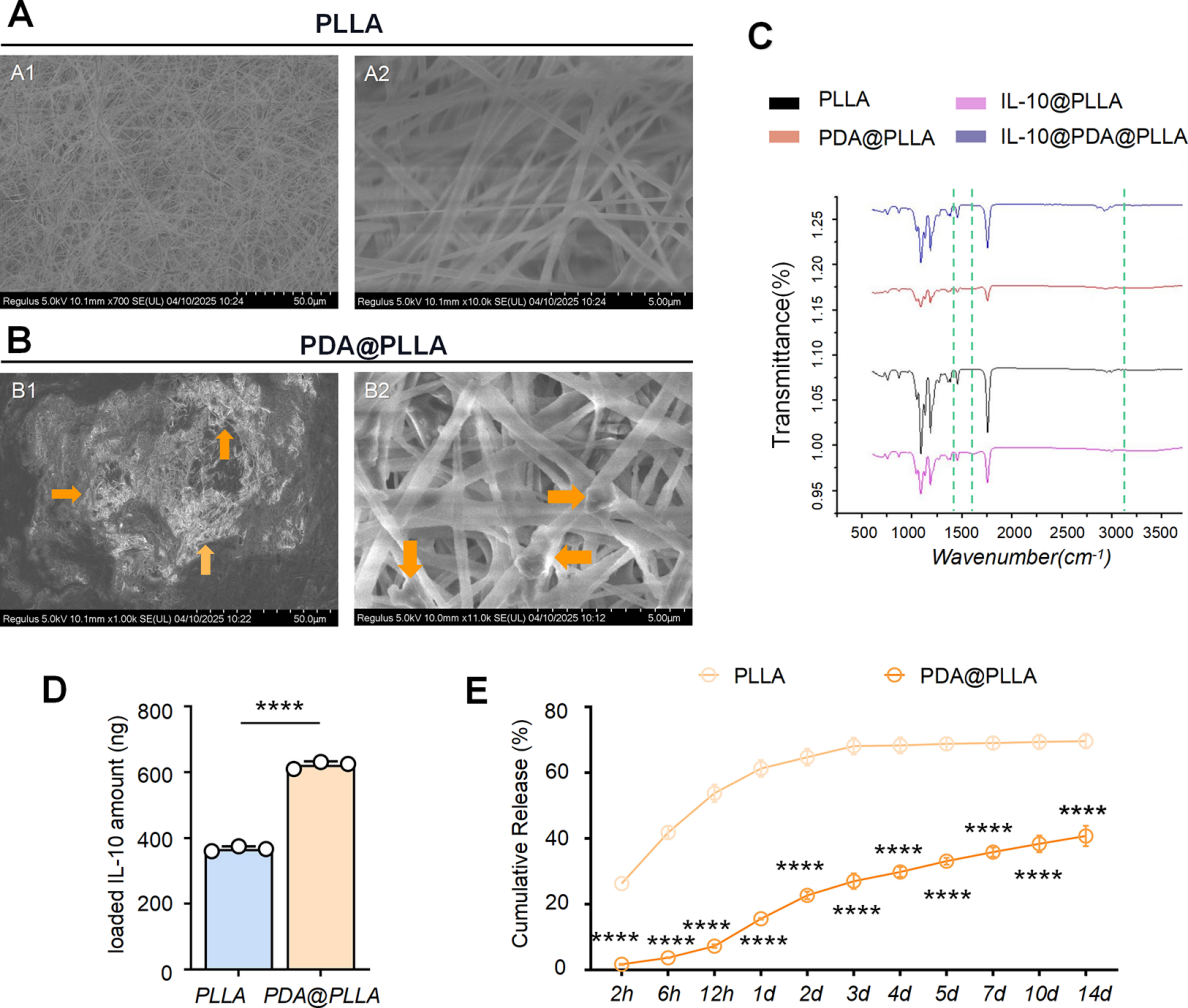
Characteristics of PDA@PLLA nanofibrous membranes and controlled release of loaded IL-10. SEM images of the PLLA membrane (A) and SEM images of the PDA@PLLA membrane (B). A uniform PDA coating was observed to be adsorbed onto the PLLA surface, as indicated by the orange arrows (B1) (scale bar, 50 μm). More details are shown at higher magnification in (B2) (scale bar, 5 μm). FTIR results of the PLLA, PDA@PLLA, IL-10@PLLA, and IL-10@PDA@PLLA membranes (C). Loaded IL-10 amount (ng) (D) and release profiles of IL-10 (%) (E) in PLLA and PDA@PLLA membranes. *n* = 3, ****p < 0.0001 versus PLLA.

### Loading and Controlled Release of IL-10 from the Nanofibers

An ELISA kit was used to evaluate the function of the PDA coating in terms of both loading and controlled release. The results revealed that after incubation, the amount of IL-10 was 367.08 ± 7.50 ng in the PLLA membrane compared with 622.41 ± 11.03 ng in the PDA@PLLA membrane, as shown in Figure. [Fig fig1]D. It is obvious that the PDA coating enhanced the loading ability of IL-10. The cumulative curve (Figure. [Fig fig1]E) showed that there was a burst with 61.29% of the total IL-10 released from the PLLA membrane on the first day, and the release was subsequently slowed, with approximately 68.10% of the total release after 3 days. In contrast, there was less of an initial burst release with 15.57% of the total IL-10 from the PDA@PLLA membrane on the first day. There was a brief burst release on the second and third day, and then the release rate slowed down; sustained release could still be observed afterward. The total release was approximately 40.75% of the total IL-10. The above results indicated that controlled release could be achieved with the help of the PDA coating. The PLLA membrane had more advantages than the PDA@PLLA membrane in terms of short-term explosive release.

### Mechanical Hypersensitivity and Neuroinflammation Developed in the SNL and SCI Models

First, we constructed stable SNL and SCI models in rats. Consistent with earlier reports, SNL induced prompt and long-lasting mechanical hypersensitivity in the left hind paw. Behavioral testing showed a sustained decrease in ipsilateral PWT from day 3 to day 21 versus sham-operated controls (Figure 2A), indicative of evolving neuropathic pain. On the other hand, SNL surgery did not affect pain sensitivity of the contralateral hind paw (Figure 2B). Likewise, T13 unilateral hemitransection elicited an immediate and persistent below-level mechanical hypersensitivity in both sides of hind paws. Compared with sham controls, PWT was significantly reduced from day 5 to day 35 in the ipsilateral (Figure 2C) and contralateral (Figure 2D) paws, documenting the establishment of chronic below-level central neuropathic pain.

**Figure 2:**
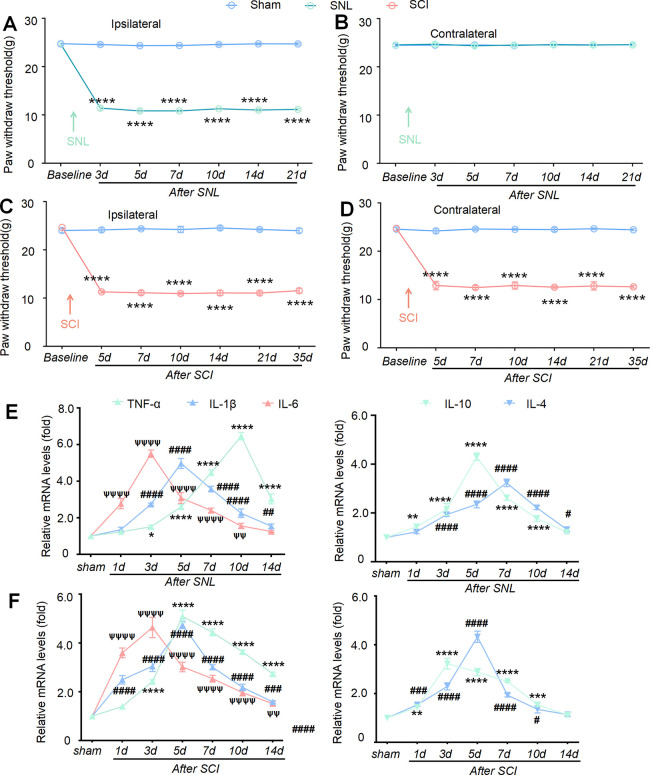
Neuropathic pain and neuroinflammation in rats after spinal nerve ligation and spinal cord hemisection injury. L5 spinal nerve ligation induced peripheral mechanical hypersensitivity in the left hind paw of rats. The PWT in the ipsilateral paw decreased from 3 to 21 days in the SNL model compared with the sham group (A), while the PWT in the contralateral paw was not affected (B). T13 spinal hemisection injury also led to rapid and persistent central mechanical hypersensitivity in both hind paws of rats. The PWT decreased from 5 to 35 days in both ipsilateral paws (C) and contralateral paws (D) compared with the sham group. Data are presented as the mean ± SEM. ****p < 0.0001 versus sham group. The mRNA levels of pro-inflammatory cytokines such as TNF-α, IL-1β, and IL-6, as well as anti-inflammatory cytokines such as IL-10 and IL-4, were significantly upregulated following SNL (E) and SCI (F). For TNF-α and IL-10, *p < 0.05, **p < 0.01, ***p < 0.001, and ****p < 0.0001 versus sham. For IL-1β and IL-4, #*p* < 0.05, ##*p* < 0.01, ###*p* < 0.001, and ####*p* < 0.0001 versus sham. For IL-6, ψψ*p* < 0.01 and ψψψψ*p* < 0.0001 versus sham.

Neuroinflammation is considered an important component of the central sensitization mechanism in neuropathic pain. We examined neuroinflammation development by checking the mRNA level of inflammatory cytokines in the spinal cord after SNL treatment. Real-time PCR results showed that SNL induced a rapid increase in the expression of TNF-α, IL-1β, and IL-6 (Figure 2E). Interestingly, the mRNA expression of TNF-α began to rise at day 3, peaked at day 10, and then gradually decreased. Similarly, the peak levels of IL-6 and IL-1β occurred at days 3 and 5, respectively. Likewise, SNL induced a gradual upregulation of the mRNA levels of anti-inflammatory cytokines including IL-10 and IL-4. However, the expression of IL-10 rapidly decreased, and the level had essentially returned to normal at 10 days after SNL, as shown in Figure 2E. We also examined the expression profile of inflammatory cytokines after SCI. The results showed that SCI induced a rapid increase in the expressions of the pro-inflammatory and anti-inflammatory cytokines (Figure 2F). It is worth noting that the elevation of the IL-10 mRNA level was only sustained for 3 days before it began to decline, as shown in Figure 2F.

### Expression of IL-10 in the Spinal Cord Was Elevated Following SNL and SCI

Next, we detected the spatiotemporal profile of IL-10 expression in the spinal cord after SNL and SCI. Western blot analysis revealed that SNL induced an initial elevation of the IL-10 protein at day 3, which then peaked on day 7 and rapidly declined. Correspondingly, the regulation of IL-10 expression was found after SCI, and this upregulation started on day 7, was maintained at a relatively high expression level from days 10 to 21, and then declined (Figure 3A). Statistical analysis confirmed these phenomena, as shown in Figure 3B. IHC analysis revealed a low IL-10 fluorescence intensity in the SDH of the sham group (Figure 3C1,C4). In contrast, IL-10 expression was significantly increased in the SDH following SNL and SCI. The highest levels were observed on the ipsilateral side on days 7 and 10 (Figure 3C3,C6), with only a slight increase on the contralateral side (Figure 3C2,C5). Quantitative analysis confirmed these changes, as shown in Figure 3D. These results indicate that the release of IL-10 can be induced in the spinal cord following SNL and SCI, with predominant expression in the ipsilateral SDH, but cannot maintain its effect over time.

**Figure 3:**
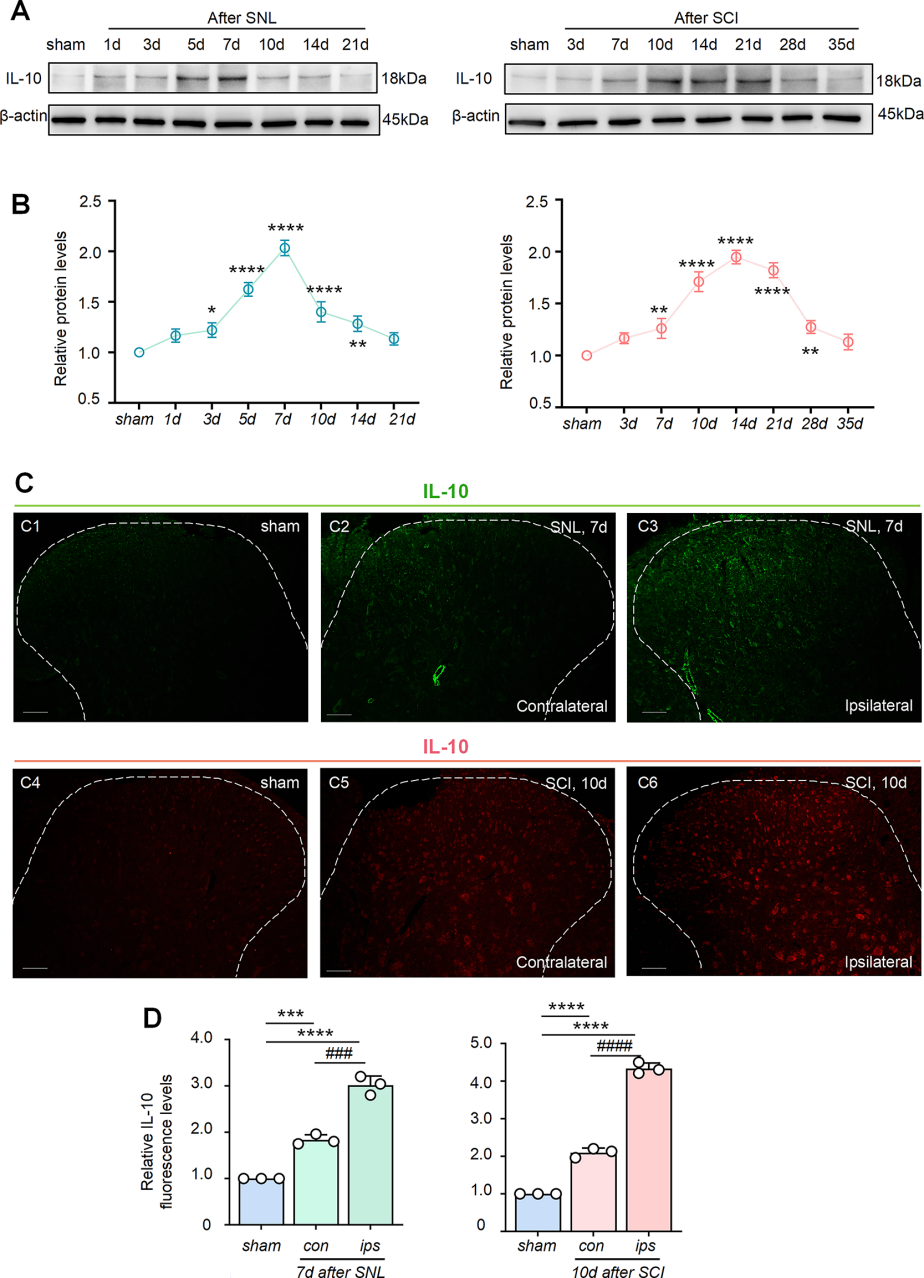
Expression and distribution of IL-10 in the spinal cord following SNL and SCI. Western blot showing the time course of IL-10 expression in SNL and SCI rats (A). SNL induced enhanced IL-10 levels in ipsilateral L4/5 spinal cord segments from days 3 to 14 after surgery, and SCI led to elevated IL-10 levels from days 7 to 28 after surgery (B). Data are presented as the mean ± SEM. *p < 0.05, **p < 0.01, and ****p < 0.0001 versus sham group. Immunofluorescence (C) showing the distribution of S100A4 (green) protein after SNL surgery in the L4/5 SDH on POD 7 (C1–C3) (scale bar, 500 μm) and the distribution of S100A4 (red) protein after SCI surgery in the L4/5 SDH on POD 10 (C4–C6) (scale bar, 500 μm). The data summary further confirmed the raised IL-10 expression after surgery, especially in the ipsilateral paw side (D). Data are presented as the mean ± SEM. ***p < 0.001 and ****p < 0.0001 versus sham. ###*p* < 0.001 and ####*p* < 0.0001 ipsilateral versus contralateral.

### IL-10-Loaded PDA@PLLA Nanofibrous Membrane Increased the Expression of IL-10 In Vivo after SNL and SCI

We further explored whether the IL-10-loaded PDA@PLLA nanofibrous membrane could sustainably release IL-10 in vivo. The rats were implanted with the IL-10-loaded PDA@PLLA nanofibrous membrane right after SNL or SCI surgery, and the L4/5 spinal cord segment was collected. WB analysis revealed a significantly higher level of IL-10 expression in the PDA@PLLA nanofibrous membrane treatment group, compared with the SNL and SCI groups at 7 days after surgery, respectively (Figure 4B). A similar trend was also observed on postoperative day 14 (Figure 4B). Semiquantitative analysis confirmed this, as shown in Figure 4C. These results indicated that the IL-10-loaded PDA@PLLA nanofibrous membrane can release the IL-10 after SNL or SCI in vivo and may further exert its anti-inflammatory function.

**Figure 4:**
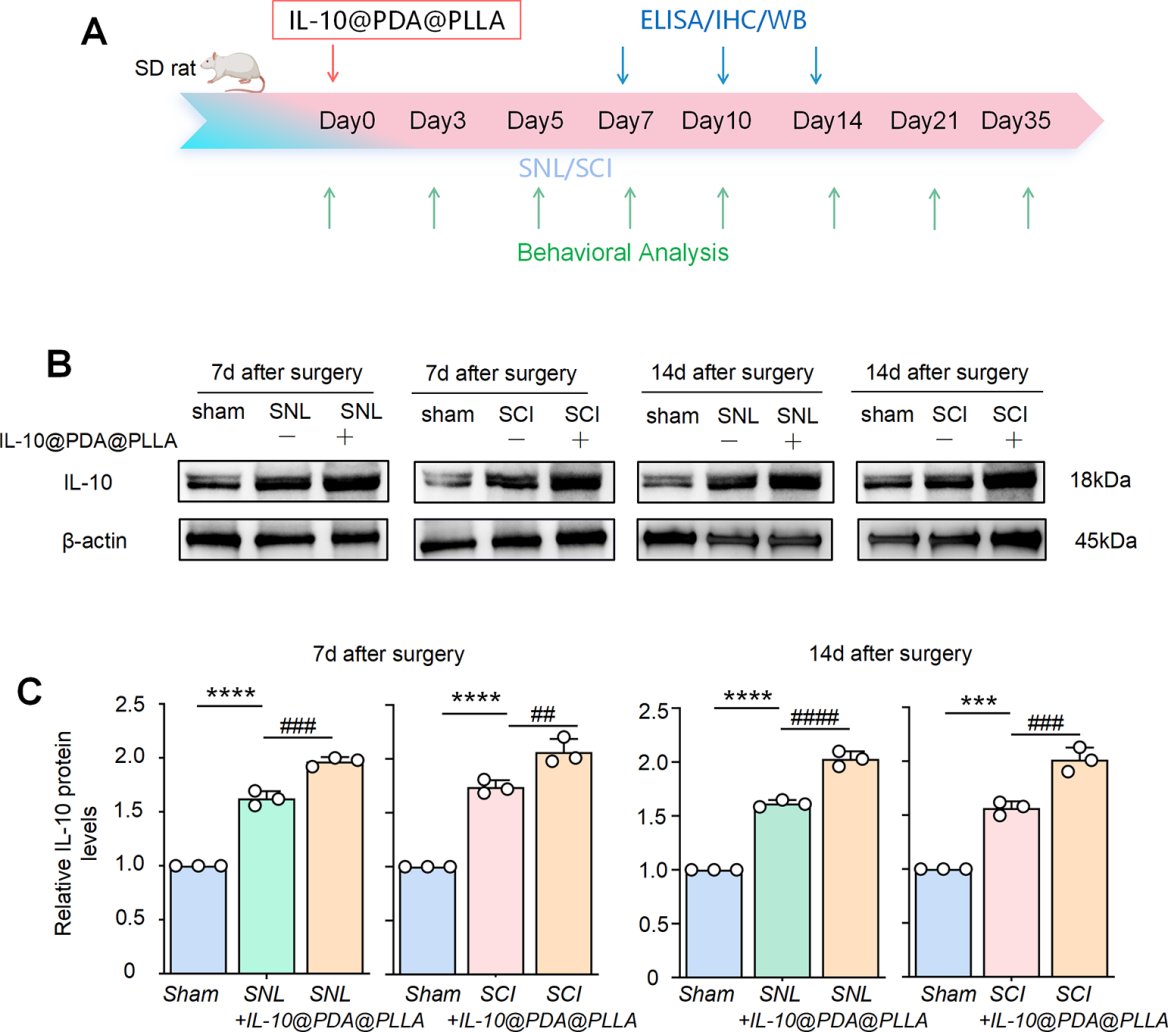
IL-10-loaded PDA@PLLA nanofibrous membrane increased the expression of IL-10 in vivo. Schematic representation of the experimental timeline, including WB, IHC, ELISA, and behavioral analyses, following the implantation of the IL-10-loaded PDA@PLLA nanofibrous membrane (A). WB analysis revealed that the PDA@PLLA nanofibrous membrane induced significantly higher levels of IL-10 expression at day 7 postsurgery in SNL and SCI rats (B). Similarly, upregulation of IL-10 was observed on day 14 postsurgery in both SNL and SCI rats after treatment with the PDA@PLLA nanofibrous membrane. The data summary further confirmed this (C). Data are presented as the mean ± SEM. ***p < 0.001 and ****p < 0.0001 versus sham. ##*p* < 0.01, ###*p* < 0.001, and ####*p* < 0.0001 versus SNL/SCI+IL-10@PDA@PLLA.

### IL-10-Loaded PDA@PLLA Nanofibrous Membrane Suppressed Central Sensitization of the SDH after SNL and SCI

Central sensitization of the SDH, including neuronal excitability, neuroglia activation, and neuroinflammation, is the major regulatory mechanism of neuropathic pain (NP). Therefore, we investigated whether implantation of the IL-10-loaded PDA@PLLA nanofibrous membrane could regulate central sensitization mechanisms.

In our IHC experiment, SCI induced significant increases in GFAP, iba-1, and c-Fos fluorescence in the ipsilateral SDH 7 days after SNL (Figure 5A2,B2,C2), compared with the sham group (Figure 5A1,B1,C1). However, the implantation of the PDA@PLLA nanofibrous membrane significantly decreased the enhanced fluorescence intensity of GFAP, iba-1, and c-Fos (Figure 5 A3,B3,C3). Semiquantitative analysis further confirmed this, as shown in Figure 5D.

**Figure 5:**
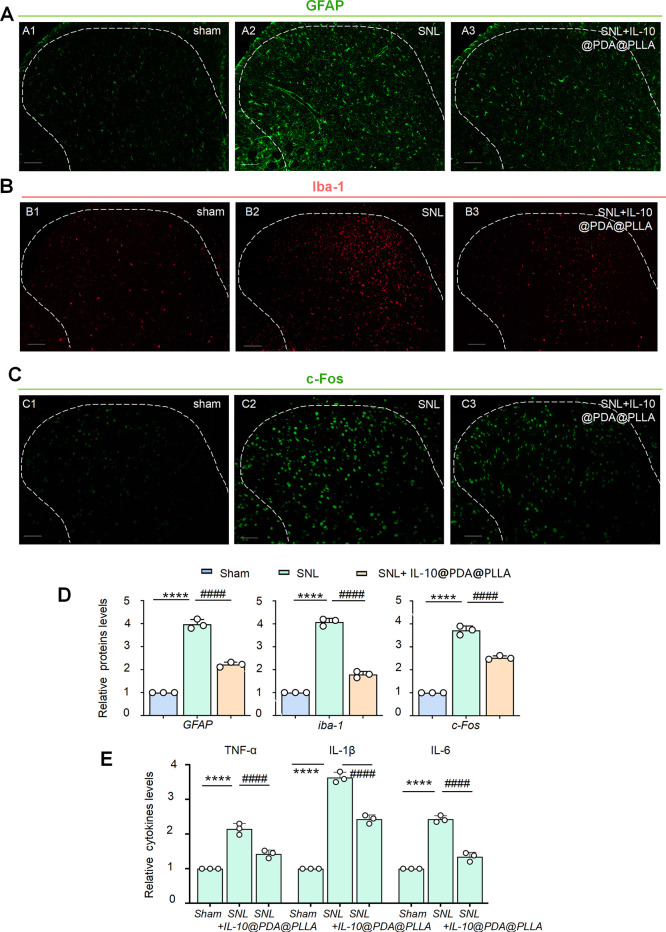
IL-10-loaded PDA@PLLA nanofibrous membrane suppressed neuronal excitability, neuroglia activation, and neuroinflammation after SNL. Fluorescence images confirmed that astrocytes were remarkably activated after SNL, and the fluorescence intensity of GFAP (green) was much more higher than in the sham group (A1 and A2) (scale bar, 500 μm). The implantation of the IL-10-loaded PDA@PLLA nanofibrous membrane suppressed SNL-induced activation of astrocytes (A3) (scale bar, 500 μm). In addition, the PDA@PLLA nanofibrous membrane blocked microglial activation (iba-1, red) and neuronal excitability (c-Fos, green) after SNL (B and C) (scale bar, 500 μm). The data summary further confirmed these results (D). ****p < 0.0001 SNL versus sham. ####*p* < 0.0001 SNL versus SNL+IL-10@PDA@PLLA. Moreover, ELISA data confirmed that pro-inflammatory cytokines, including TNF-α, IL-6, and IL-1β were significantly upregulated after SNL compared with those in the sham group, and their protein levels were reduced after treatment with the PDA@PLLA nanofibrous membrane (E). ****p < 0.0001 SNL versus sham. ####*p* < 0.0001 SNL versus SNL+IL-10@PDA@PLLA.

Besides, ELISA revealed a marked SNL-induced elevation of the pro-inflammatory cytokines TNF-α, IL-6, and IL-1β versus sham levels, while application of the PDA@PLLA nanofibrous membrane significantly reduced their spinal content (Figure 5E).

Likewise, SCI induced significant increases in GFAP, iba-1, and c-Fos fluorescence in the ipsilateral SDH 10 days after surgery, while the PDA@PLLA nanofibrous membrane with the sustained release of IL-10 could partially block the elevated fluorescence intensity caused by SCI (Figure 6A–C). Semiquantitative analysis further confirmed this, as shown in Figure 6D. Additionally, the ELISA test confirmed lower levels of pro-inflammatory cytokines after treatment with the PDA@PLLA nanofibrous membrane (Figure 6E). Therefore, the IL-10-releasing PDA@PLLA nanofibrous membrane may regulate the central sensitization of the SDH by gradually releasing the anti-inflammatory cytokine IL-10.

**Figure 6:**
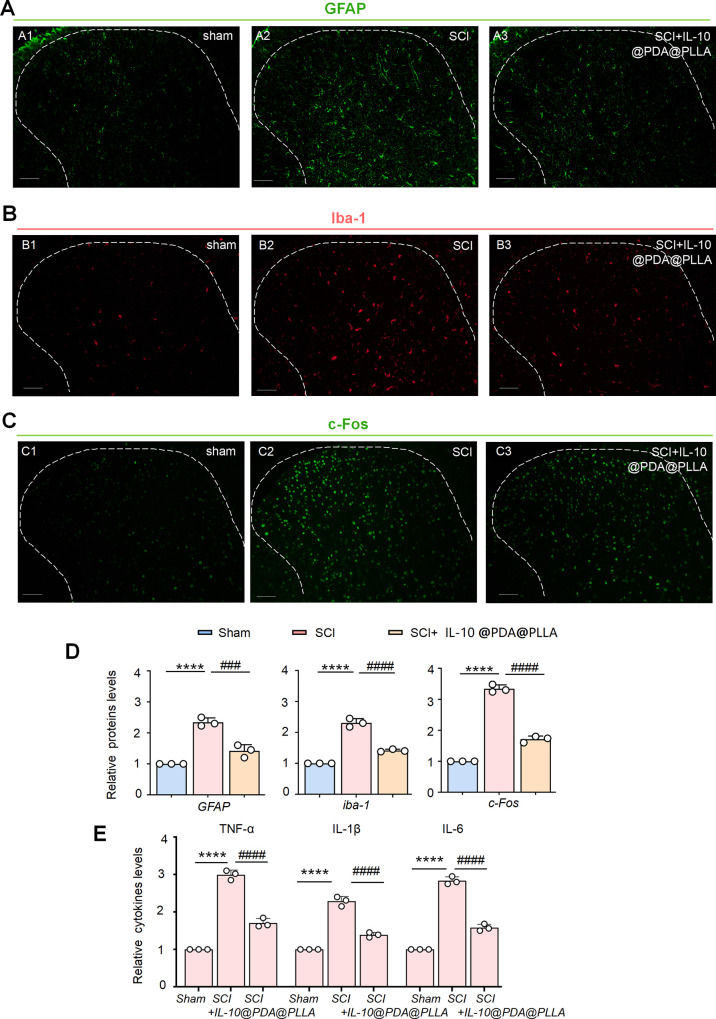
IL-10-loaded PDA@PLLA nanofibrous membrane suppressed neuronal excitability, neuroglia activation, and neuroinflammation after SCI. Similarly, immunofluorescence analysis confirmed that the heightened fluorescence intensity of GFAP, iba-1, and c-Fos after SCI was suppressed after implantation of the IL-10-loaded PDA@PLLA nanofibrous membrane (A–C). The data summary further confirmed these results (D). ****p < 0.0001 SNL versus sham. ####*p* < 0.0001 SCI versus SCI+IL-10@PDA@PLLA. ELISA assay confirmed that the elevated pro-inflammatory cytokine protein levels in SCI rats were significantly decreased after treatment with the PDA@PLLA nanofibrous membrane (E). ****p < 0.0001 SCI versus sham. ####*p* < 0.0001 SCI versus SCI+IL-10@PDA@PLLA.

### PDA@PLLA Nanofibrous Membrane Loaded IL-10 Attenuates NP Following SNL and SCI

Finally, we determined that the PDA@PLLA nanofibrous membrane loaded IL-10 contributes to the regulation of pain hypersensitivity in rats by behavioral tests. The rats were treated with the IL-10@PDA@PLLA nanofibrous membrane right after SNL and SCI. Mechanical hypersensitivity was markedly ameliorated (Figure 7A,C,D). Ipsilateral paw-withdrawal thresholds remained significantly lower at 3, 5, 7, 10, and 14 days after SNL in the PDA@PLLA nanofibrous membrane-treated group, and the antihyperalgesic effect remained detectable up to postinjury day 21 (Figure 7A). On the other hand, treatment left contralateral hind paw mechanical thresholds unchanged (Figure 7B). We also explored whether implantation of the PDA@PLLA nanofibrous membrane could similarly mitigate SCI-evoked mechanical hypersensitivity. The mean paw mechanical withdrawal thresholds on both the ipsilateral side and contralateral side were significantly decreased at 5, 7, 10, 14, and 21 days after SCI in the PDA@PLLA nanofibrous membrane-treated group, The improvements in hyperalgesia continued to be evident up to 35 days after injury, as shown in Figure 7C,D. These results demonstrate that the IL-10-loaded PDA@PLLA nanofibrous membrane alleviates peripheral neuropathic pain in SNL rats and central neuropathic pain in SCI rats.

**Figure 7:**
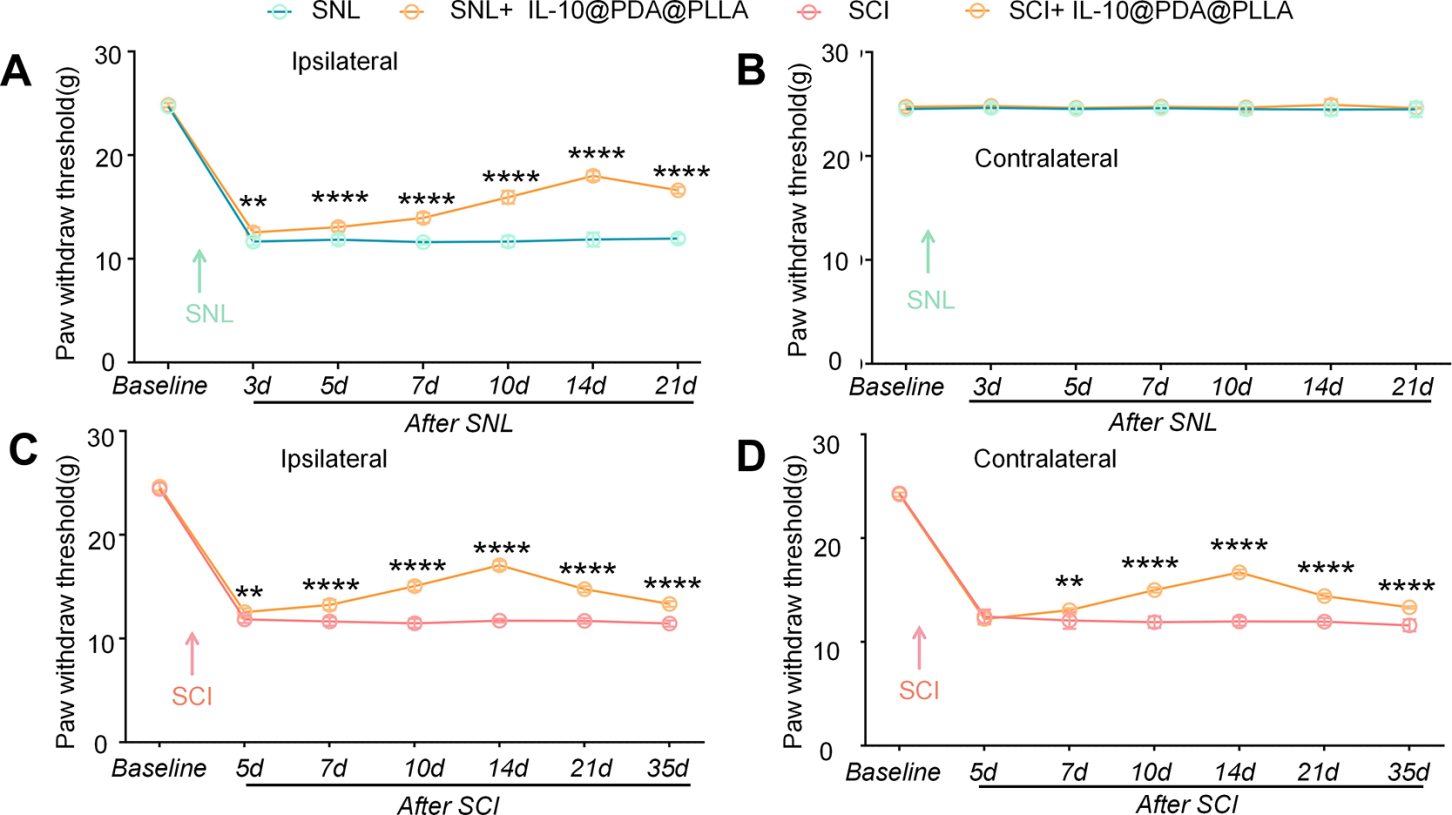
PDA@PLLA nanofibrous membrane loaded IL-10 attenuates mechanical pain following SNL and SCI. The implantation of the IL-10-loaded PDA@PLLA nanofibrous membrane partially suppressed peripheral mechanical hypersensitivity in the ipsilateral hind paw for up to 21 days after SNL, and the effect was observed 3 days after surgery (A); the implantation did not affect the PWT in the contralateral hind paw (B). Data are presented as the mean ± SEM. **p < 0.01 and ****p < 0.0001 SNL versus SNL+IL-10@PDA@PLLA. Similarly, treatment with the PDA@PLLA nanofibrous membrane attenuated the development of central mechanical hypersensitivity in SCI rats. The PWT in the ipsilateral hind paws increased from day 5 and remained elevated for up to 21 days after SCI (C). In contrast, the PWT in the contralateral hind paws began to increase from day 7 and remained elevated until day 21 following SCI (D). Data are presented as the mean ± SEM. **p < 0.01 and ****p < 0.0001 SCI versus SCI+IL-10@PDA@PLLA.

## Discussion

Neuropathic pain usually brings a serious physiological and psychological burden to patients. However, there is no clear effective treatment in clinic. Existing drug therapies have difficultly meeting the requirements for the sustained relief of symptoms in all patients, and invasive physical therapy is a long way from widespread clinical use.^36^ Therefore, the research target in its mechanisms and innovative treatment methods has significant clinical value. In our experiment, we constructed a neuropathic pain model using two surgical procedures, spinal nerve ligation (SNL) and spinal cord injury (SCI). We measured the paw-withdrawal threshold and discovered that the model rats were more sensitive to painful stimulus compared to the control. We found that persistent peripheral neuropathic pain occurred in the ipsilateral hind paws of rats in the SNL model, and below-level neuropathic pain was observed in both hind paws of rats in SCI model, which is consistent with the clinical manifestations of neuropathic pain.^37^

IL-10 has been shown to be involved in the regulation of neuropathic pain through an anti-inflammatory effect. Aghili discovered that mirtazapine could attenuate mechanical, thermal, and cold allodynia post-SCI via neuroinflammation modulation, partially through increasing the anti-inflammatory cytokine IL-10 expression level.^38^ Similarly, IL-10 was deeply involved in the inflammatory response of microglia induced by IL-35 in vitro, which was closely related to the effects of IL-35 on peripheral pain behaviors following chronic constriction injury in male mice.^39^ Consistently, the pro-inflammatory factors and anti-inflammatory factors including IL-10 showed time-dependent changes in our neuropathic pain models. Furthermore, IL-10 expressed in the spinal dorsal horn significantly increased. These results suggest the development of neuroinflammation after SNL and SCI. Notably, upon reaching its peak in the early stage, IL-10 levels began to gradually decline. Although the exact mechanism is still unclear, we suppose that continuously increasing endogenous IL-10 helps to antagonize neuropathic pain.

According to our previous work, we introduced electrospun membranes to achieve the loading and release of interleukin-10. PLLA was preferred as the scaffold because of its good biocompatibility. Studies have shown that PLLA can eventually be absorbed in the body. Rambhia adopted the PLLA nanofiber scaffold as the carrier for releasing osteogenic proteins in his research and achieved the expected results.^40^ A recent study has shown that novel poly­(d,l-lactic-*co*-glycolic acid) (PLGA) nanoparticles delivering anti-inflammatory cytokines, including IL-10, could be a new therapeutic strategy for neuropathic pain.^41^ Electrospinning can process polymer solutions into nanoscale fiber films. The electrospun membrane has high specific surface area and porosity, which are both beneficial for loading factors and nutrient delivery.^42^ In this study, we fabricated a PLLA membrane through electrospinning. It appeared as crisscrossing fibers under SEM. We introduced a PDA coating in order to increase the loading rate of IL-10 on the PLLA membrane, which is indeed consistent with our results. Therefore, a novel artificial material was designed to maintain local IL-10 concentrations, which was successfully achieved on the basis of our FTIR results. Besides, we also confirmed that both PLLA and PDA@PLLA membranes could load and release the IL-10 cytokine, but PDA@PLLA membranes could achieve a more prolonged controlled release. It could still release a large proportion of loaded IL-10 after 14 days. We speculated that the PDA coating enhanced the adsorption effect of IL-10 on the PLLA film and made it more stable, resulting in difficulty in release. More importantly, IL-10 cytokine levels in the spinal cord were significantly upregulated after the implantation of PDA@PLLA nanofibers in rats, in both SNL and SCI models. In summary, the combination of IL-10 and PDA@PLLA nanofibers provides a novel implantable cytokine delivery system to continuously exert anti-inflammatory effects under the state of neuropathic pain.

Accumulating evidence suggests that central sensitization mechanisms including neuronal excitability, glial cell activation, and neuroinflammation participate in the regulation of neuropathic pain.^43–45^ In our research, immunofluorescence results confirmed that the expression of c-Fos, GFAP, and iba-1 in the spinal dorsal horn was significantly increased after SNL and SCI, indicating neuronal responsiveness and glial activation after surgery, while the implantation of the IL-10-loaded PDA@PLLA membrane decreased these changes. Moreover, our ELISA data validated that the elevated protein expression of pro-inflammatory cytokines including TNF-α, IL-6, and IL-1β was reduced by treatment with the IL-10-loaded nanofibrous membrane. In brief, we believed that this PDA@PLLA membrane with controlled release of IL-10 could regulate the central sensitization mechanisms, including neuroinflammation, which is the principal mechanism of neuropathic pain.

More importantly, we observed that the implantation of PDA@PLLA nanofibers could continuously increase the mechanical pain threshold of the hind paws of rats. Collectively, we speculate that the IL-10@PDA@PLLA nanofibrous membrane could maintain the local concentration of IL-10 through sustained release and further exert antineuroinflammatory effects in the spinal dorsal horn and relieve neuropathic pain following SNL and SCI eventually.

## Conclusions

In conclusion, this study demonstrated that IL-10-loaded PDA@PLLA nanofibers attenuate peripheral and central neuropathic pain in rats. Mechanistically, it may achieve these effects by the controlled release of cytokine IL-10 and antagonizing neuroinflammation in the spinal cord. Moreover, we revealed that treatment with the IL-10-loaded PDA@PLLA nanofibrous membrane could regulate central sensitization mechanisms in the SDH. Our findings elucidate that IL-10@PDA@PLLA nanofibers provide an innovative implantable system for managing the neuroinflammatory process and may emerge as a potential therapeutic for alleviating chronic neuropathic pain.

## Supplementary Material



## Notes

Primers for real-time PCR (PDF)
