# Supplementary material for: Polydopamine-Coated Poly(l‑lactide) Nanofibers with Controlled Release of IL-10 for Effective Management of Peripheral and Central Neuropathic Pain in Rats
Source: ACS Biomater Sci Eng. 2025 Nov 13;11(12):7269–81. doi: 10.1021/acsbiomaterials.5c01638 (PMC12690514; doi:10.1021/acsbiomaterials.5c01638)
Supplement: Supplementary file 1 [file ab5c01638_si_001.pdf]

Table 1. Primers for real-time PCR

Primer	Forward sequence	Reverse sequence
TNF-α	CCACGCTCTTCTGTCTACTG	GCTACGGGCTTGTCACTC
IL-6	TGCCTTGGGACTGAT	TTGCCATTGCACAACTCT
IL-1β	TGTGATGTTCCCATTAGAC	AATACCACTTGTTGGCTTA
IL-10	AGGGTTACTTGGGTTGCC	GGGTCTTCAGCTTCTCTCC
IL-4	CAAGGAACACCACGGAGAA	AGCACGGAGGTACATCACG
GAPDH	CCTGGAGAAACCTGCCAAGTA	TCATACCAGGAAATGAGCTTGAC

209x120mm (192 x 192 DPI)
